# Stacked SiGe nanosheets p-FET for Sub-3 nm logic applications

**DOI:** 10.1038/s41598-023-36614-2

**Published:** 2023-06-09

**Authors:** Chun-Lin Chu, Shu-Han Hsu, Wei-Yuan Chang, Guang-Li Luo, Szu-Hung Chen

**Affiliations:** 1 Taiwan Semiconductor Research Institute, NARL, Hsinchu, Taiwan; 2 Functional Advanced Materials Engineering Research Center (FAME), Sirindhorn International Institute of Technology, Thammasat University, Pathum Thani, 12120 Thailand; 3 School of Integrated Science and Innovation-ISI, Sirindhorn International Institute of Technology (SIIT), Thammasat University, Pathum Thani, 12120 Thailand

**Keywords:** Materials science, Nanoscience and technology

## Abstract

The fabrication of vertically stacked SiGe nanosheet (NS) field-effect transistors (FETs) was demonstrated in this study. The key process technologies involved in this device fabrication are low pressure chemical vapor deposition SiGe/Si multilayer epitaxy, selective etching of Si layers over SiGe layers using tetramethyl-ammonium-hydroxide wet solution, and atomic layer deposition of Y_2_O_3_ gate dielectric. For the fabricated stacked SiGe NS p-GAAFETs with a gate length of 90 nm, I_ON_/I_OFF_ ratio of around 5.0 × 10^5^ and subthreshold swing of 75 mV/dec were confirmed via electrical measurements. Moreover, owing to its high quality of Y_2_O_3_ gate dielectric, the device showed a very small drain-induced barrier-lowering phenomenon. These designs can improve the gate controllability of channel and device characteristics.

## Introduction

In response to market demands, CMOS technologies continuously aim to scale down the device dimension as much as possible. Nevertheless, miniature Si metal–oxide–semiconductor field-effect transistors (MOSFETs) have encountered many fundamental physical limits; the accompanying difficulties in either developing required process technology or enhancing device performance are also increased. Simply shrinking the channel length and/or the dielectric thickness can no longer realize the excellent switching ratio, high driving capability, low leakage current, and acceptable reliability. Compared to pure Ge channel FET, SiGe channel FETs will be most likely utilized beyond 3-nm technology node because of the absence of dislocations resulting from the smaller lattice mismatch with Si^1^. Thus, rapid development in SiGe technologies is warranted, and relevant studies in this field are needed^2–13^.

Many reports regarding the stacked channel FETs have been published. However, few studies have reported on the stacked SiGe channel FETs. The main reason behind this is that selective etching of Si over SiGe is a difficult process. In this work, we fabricated vertically stacked SiGe nanosheet (NS) FET devices on cost-effective traditional Si substrates by selective etching of Si in SiGe/Si/SiGe stack structures using wet tetramethyl-ammonium-hydroxide (TMAH) solution to form stacked SiGe NS channels. The fabrication process exhibits various advantages. (1) isotropic etching without ion bombardment with extremely high Si to SiGe selective etching rate ratio; (2) better control of the composition, thickness, and spacing of SiGe channel materials; (3) low process complexity, which is in line with standard process and mass production. These advanced designs can improve the gate controllability of channel and device characteristics. As the semiconductor-related field gradually enters its physical limitations, Si/SiGe/Si/SiGe epitaxial multi-layer combined with selective etching is considered as the most likely implementation of the vertically stacked SiGe^14,15^.

In this work, selective etching of Si over SiGe was systematically studied and a complete process-flow for the formation of vertically stacked SiGe channels was developed. Recently, highly selective etchants were demonstrated in our group^1^, which enabled the fabrication of SiGe NS using a wet-etching approach. After selective etching, optimization was done to achieve high-k gate oxide solutions on p-FET channels and obtain an applicable interface quality for demonstration.

## Device fabrication

**Figure 1:**
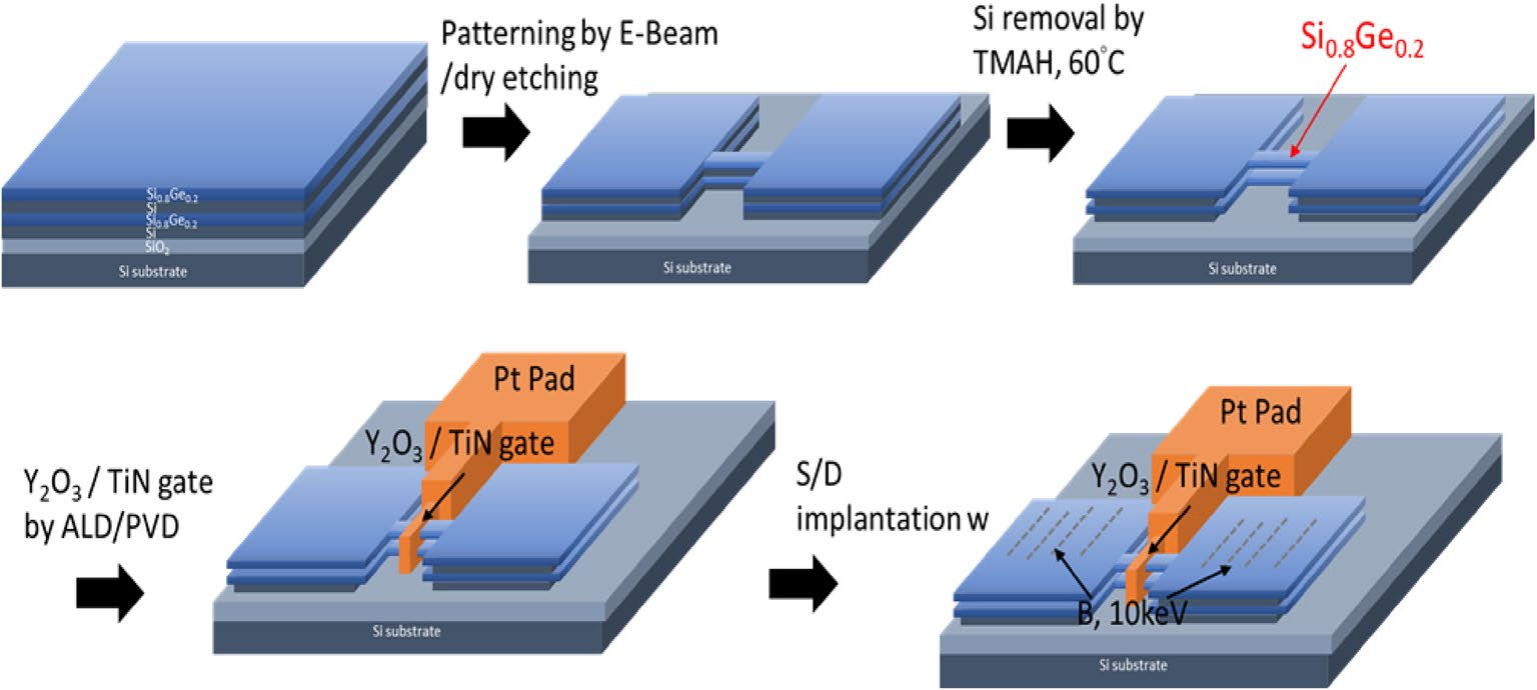
Schematic representation of SiGe p-GAA FETs device fabrication.

**Figure 2:**
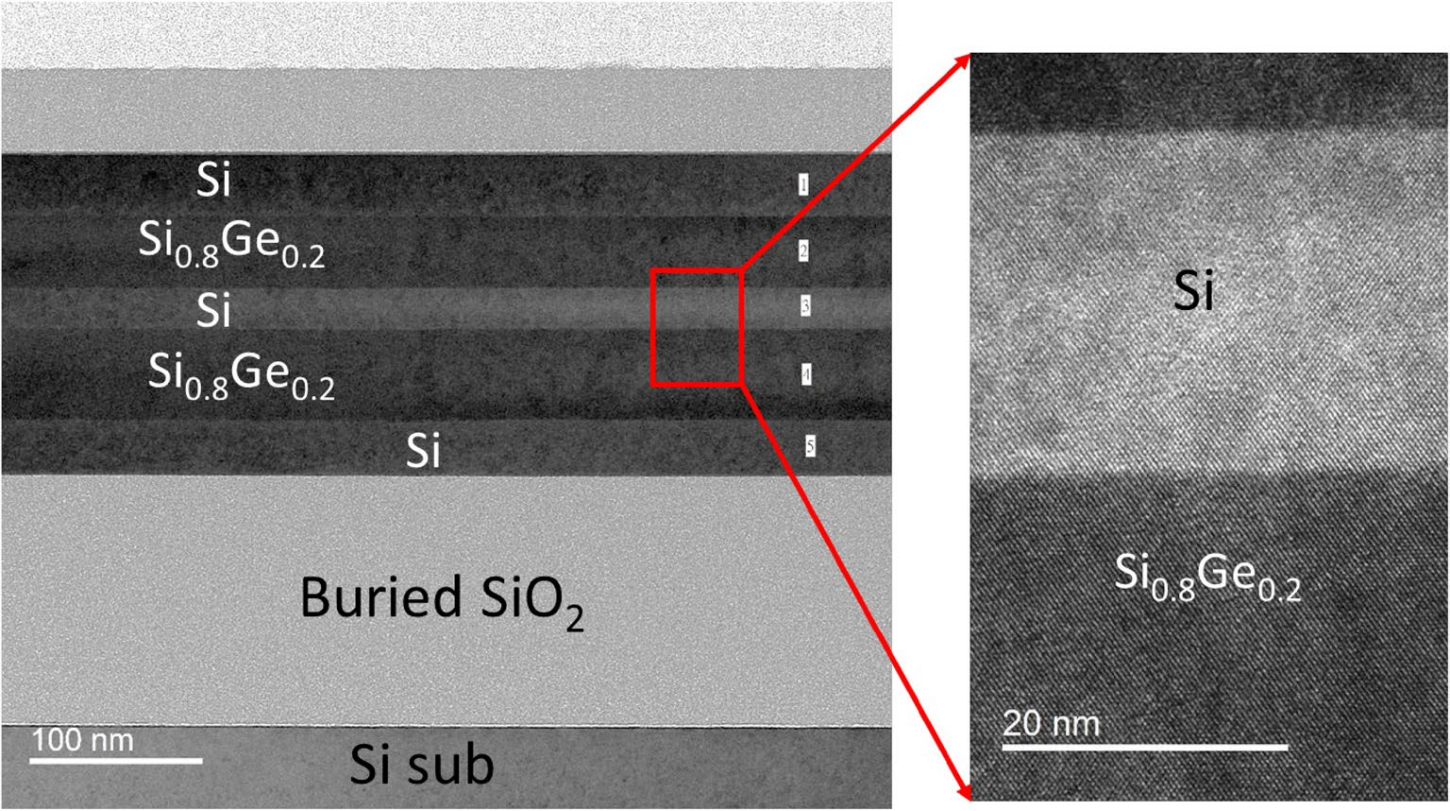
Cross-sectional TEM images of SiGe/Si multilayers.

**Figure 3:**
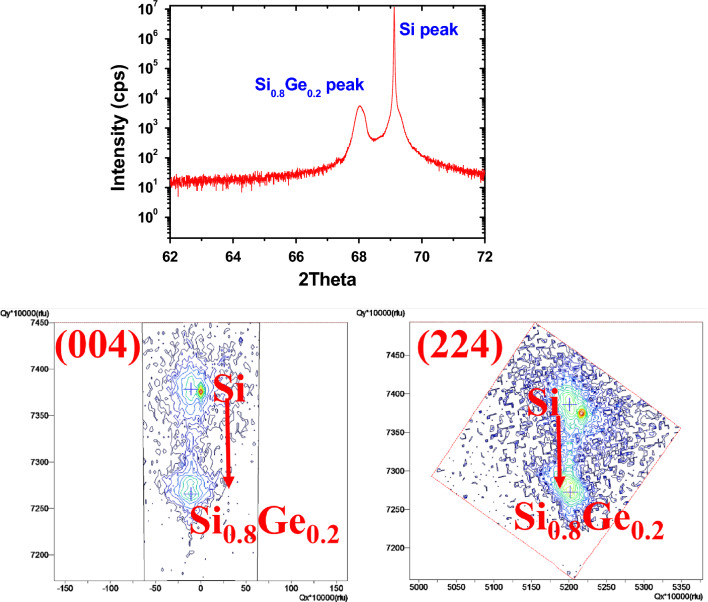
XRD rocking curve analysis and reciprocal space mapping (RSM) of SiGe/Si multilayers.

**Figure 4:**
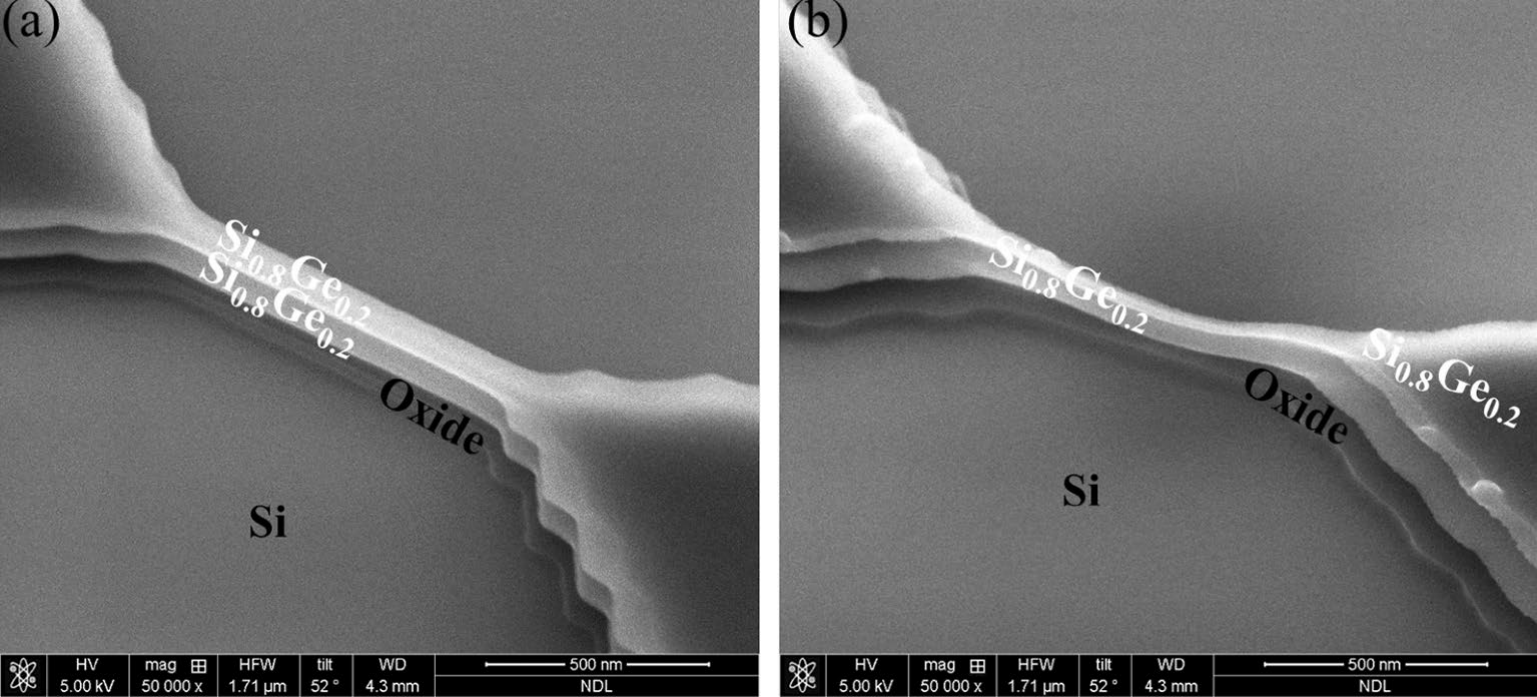
SEM images of stacked Si_0.8_Ge_0.2_ nanosheets after Si interlayers were selectively etched using TMAH solution. (**a**) Successfully formed nano-sheets. (**b**) Bending nano-sheets.

**Figure 5:**
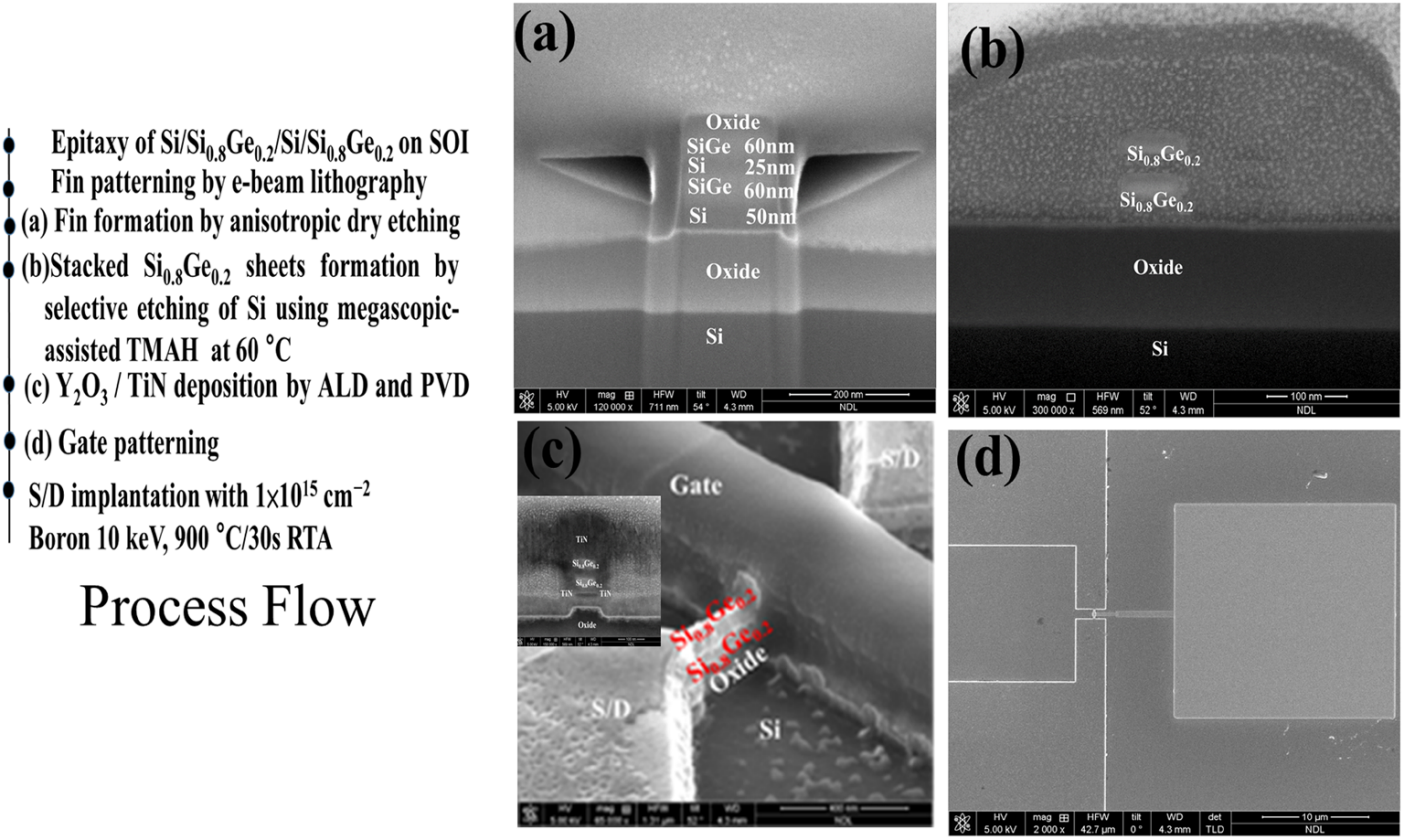
Simplified process sequence with selected cross-sectional SEM pictures from critical steps: (**a**) Key process flow of Si_0.8_Ge_0.2_ nanosheets p-FET fabrication; (**b**) a fin formation for channel and S/D extension regions; (**c**) stacked Si_0.8_Ge_0.2_ nanosheet structures formation by megasonic-agitation-assisted TMAH selective etching at 60 °C; (**d**) conformal depositions of Y_2_O_3_/TiN as gate dielectric and metal; (**e**) top view SEM image of a finished device.

The fabrication procedure of two stacked p-type SiGe NS gate-all-around field-effect transistor (p-GAA FETs) is illustrated in Fig. 1. Two periods of Si_0.8_Ge_0.2_ (60 nm)/Si (25 nm) layers were epitaxially grown on 200 mm SOI (100) substrate with a top Si thickness of 40 nm and a buried oxide thickness of 150 nm by low pressure chemical vapor deposition (LPCVD) using Dichlorosilane and GeH_4_ gases. The GeH_4_ and DCS flow rates were set to 25 and 130 sccm, respectively. When only Si layer was grown, the GeH_4_ was switched to vent mode (that is, GeH_4_ bypassed the chamber). The growth temperature and pressure were kept at 700 °C and 20 torr for all layers. A specific SiGe/Si epitaxy was employed to stack NS channels, with Si being used as a sacrificial layer that defines the suspension thickness between the channels. Figure 2 shows the transmission electron microscopy (TEM) cross-sectional images of the SiGe/Si multilayer epitaxy. The alternative layer thickness was well-controlled giving precise film thickness. X-ray diffraction (XRD) rocking curve analysis and reciprocal space mapping (RSM) in Fig. 3 show that the SiGe layers in the stacking SiGe/Si multilayers are fully strained, implying that the SiGe layers are not relaxed, and therefore no dislocations are generated. This results can also be supported by TEM images in Fig. 2. After LPCVD epitaxy, the active region of the device was defined by electron-beam lithography. Subsequently, the active region was isolated by etching Si_0.8_Ge_0.2_/Si stacked layers down to the BOX layer using Cl_2_/HBr-related etching process. The fabricated fin structures in the central area of the active region have a width of ~ 100 nm. After the fin formation, we used TMAH solution (2.38% in H_2_O) to selectively etch away Si layers and keep Si_0.8_Ge_0.2_ layers. To achieve a good selective etching between Si_0.8_Ge_0.2_/Si, megasonic agitation was used with the TMAH solution at approximately 60 °C. Figure 4 shows the stacked Si_0.8_Ge_0.2_ NS structures after selective etching. As shown in Fig. 4a, the stacked Si_0.8_Ge_0.2_ NS with a nanosheet width (W_NS_) of ~ 100 nm is released. The N_2_ blow drying process on the sample after wet-etching must be performed cautiously. Strong N2 blowing would lead to the issue of NS bending (see Fig. 4b). The resultant NS thickness is greater than 30 nm, and it is possible to obtain a thinner NS and higher W_NS_ with further optimization. Subsequently, the 3 nm high-k dielectric Y_2_O_3_ gate dielectric was deposited by atomic layer deposition (ALD) and TiN gate metal layer was deposited by PVD sputtering with a thickness of 75 nm. The deposited thickness of TiN on the sidewall was ~ 60 nm. After gate patterning, the S/D regions were formed by ion-implanted using ^11^B ions for SiGe nanosheets p-FET (1 × 10^15^ cm^−2^ at 10 keV). Activation was accomplished by annealing at 900 ℃ for 30 s. A simplified process flow with selected cross-sectional SEM pictures from critical steps is shown in Fig. 5.

## Results

**Figure 6:**
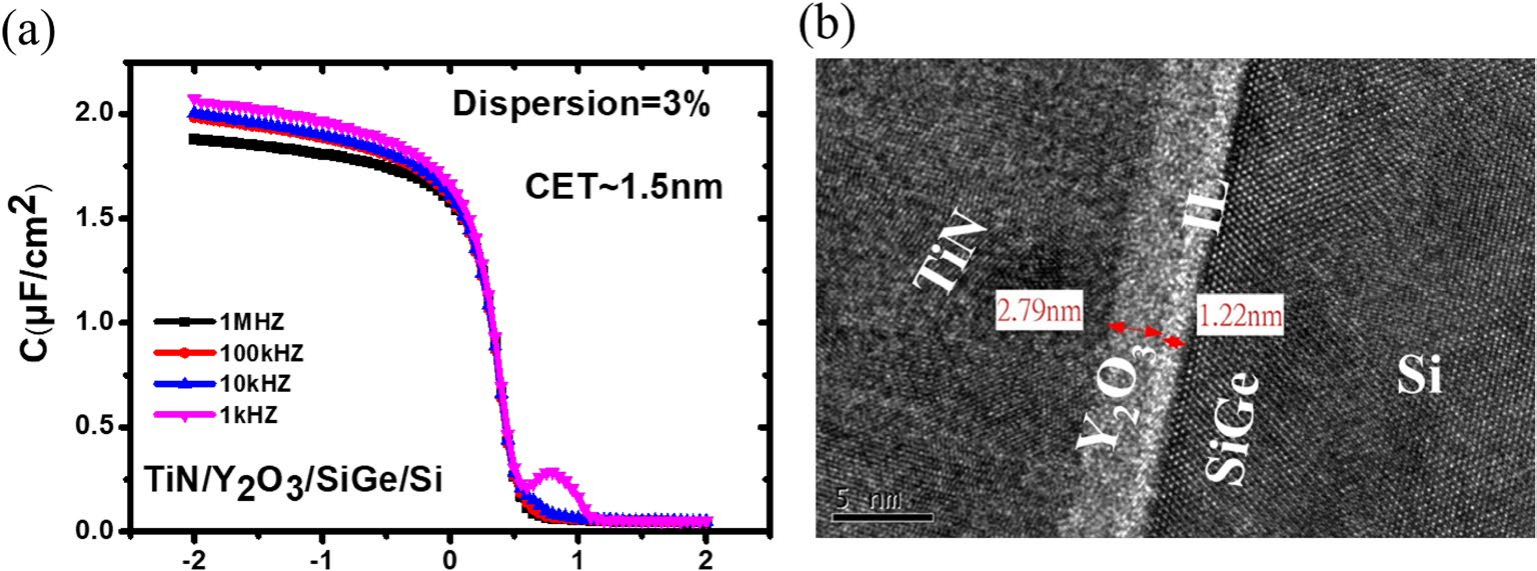
Multifrequency C–V characteristics of SiGe MOSCAPs (**a**) C–V curves from 1 kHz to 1 MHz and (**b**) Cross-sectional high-resolution transmission electron microscopy (HRTEM) images of gate stacks.

In our previous work, we had reported SiGe MOS interfacial properties using a gate stack of Y_2_O_3_ with ten cycles of TMA pre-treatment. The scalability of EOT via reduction in film thickness of Y_2_O_3_ and its effects on the properties of Si_0.8_Ge_0.2_ MOS interfaces still remained unclear^16,17^. In this work, the feasibility of EOT scaling in TiN/Y_2_O_3_ gate stacks with TMA treatment was examined. Figure 6a shows the capacitance–voltage (C–V) measurement on a control TiN/Y_2_O_3_/p-Si_0.8_Ge_0.2_ MOS capacitor, which was fabricated using the same ALD Y_2_O_3_ process as the device. The C–V curves of Si_0.8_Ge_0.2_ MOS devices exhibit only 3% frequency dispersion between 1 kHz and 1 MHz in the accumulation region at room temperature, indicating that the bulk Y_2_O_3_ was of high quality. Preliminary results on TiN/Y_2_O_3_/p-Si_0.8_Ge_0.2_ structures that underwent forming gas annealing indicate a decrease in the calculated equivalent thickness (CET), implying that ALD Y_2_O_3_ is a promising gate dielectric. Based on the measured C–V curves, the calculated equivalent thickness is ~ 1.5 nm. To characterize Y_2_O_3_ film properties and the depth profiles of each element, high resolution transmission electron microscopy (HRTEM) was performed. The TEM images and depth profiles from TiN/Y_2_O_3_ with TMA treatment are shown in Fig. 6b. A thinner interfacial layer was observed between Y_2_O_3_ and SiGe showing an amorphous characteristic. This suggests an amorphous nature of interface layers (ILs), making the crystallization of ultrathin Y_2_O_3_ films difficult. However, C–V characteristics indicated sufficient performance of this gate stack for further device fabrication.

**Figure 7:**
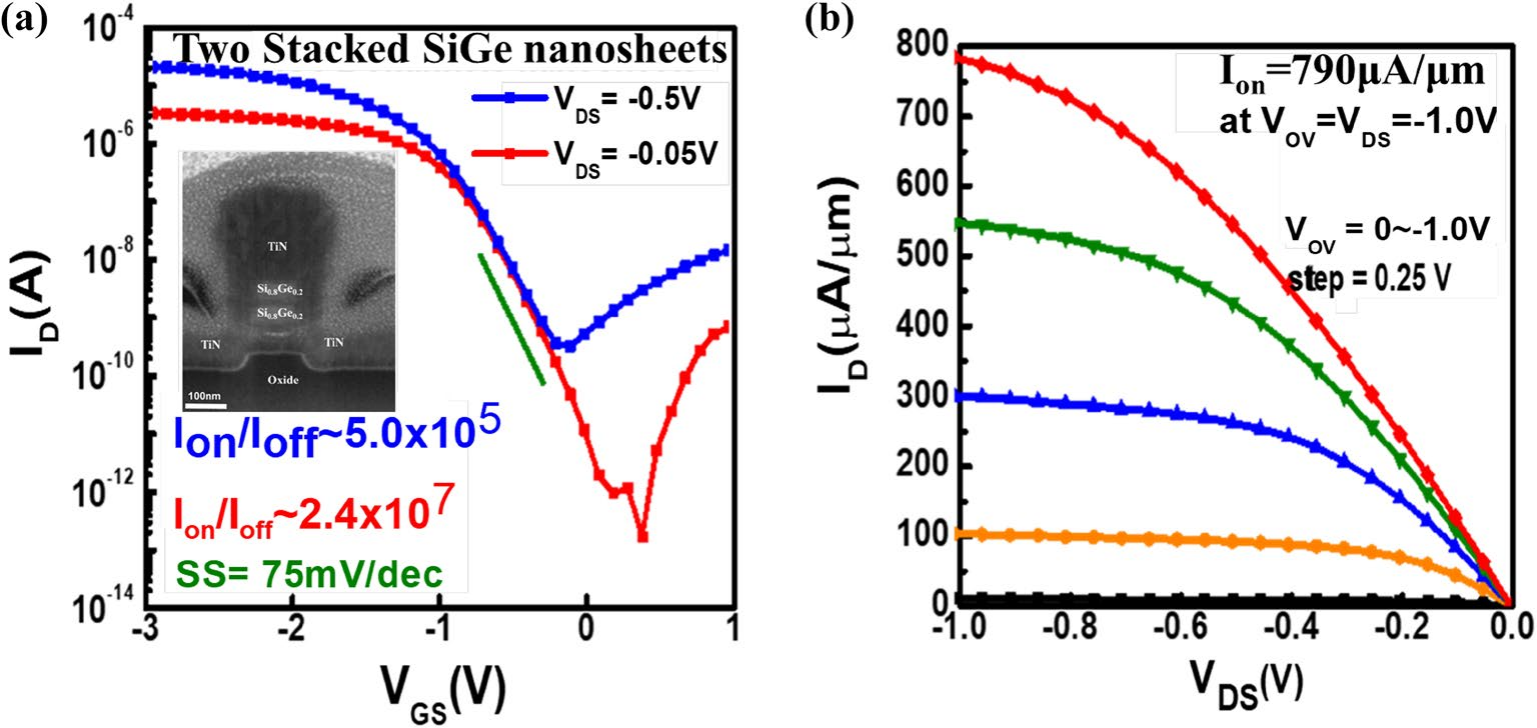
(**a**) I_D_–V_GS_ and (**b**) I_D_–V_DS_ curves for the two stacked SiGe nanosheet p-GAAFET with a gate length of 90 nm.

Finally, I_D_–V_GS_ and I_D_–V_DS_ curves for the nanosheets p-FETs are shown in Fig. 7a and b, respectively. In Fig. 7b, the gate overdrive voltage V_OV_ sweeps from 0 to 1.0 V with steps of 0.25 V. The normalized current (per footprint width of SiGe nanosheets) at V_OV_ =  − 0.5 V and V_DS_ =  − 1.0 V is approximately 790 μA/μm. The I_ON_/I_OFF_ ratios for the p-GAAFETs are approximately 1 × 10^6^. These devices show negligible drain-induced barrier lowering effects, indicating that the electrostatic control of the all-around gates on the stacked Si_0.8_Ge_0.2_-NS channels is excellent. The subthreshold swings (SS) are approximately 70 mV/dec. Table 1 shows a comparison of Si_0.8_Ge_0.2_ stacked NS GAAFETs in this study with other GAAFETs that use different stacked channels.

**Table 1:** Comparison of this study with other stacked channel GAAFETs.

Device type of GAAFETs	Si stacked NS	Si stacked NWs	SiGe stacked NS (Current work)
	pFET^13^	pFET^14^	pFET
W_NS_ or D_NW_	~ 15 nm	30 nm	100 nm
Gate length	12 nm	650 nm	90 nm
Normalization method	N/A	Perimeter of NWs	Footprint of NS
I_on_ (μA/μm)	N/A	550	790
I_on_/I_off_	~ 10^6^	~ 10^6^	~ 10^7^
SS (mV/dec)*	85	66	75
V_D_(V)/V_G_ (V)	− 0.7/− 1.2	− 1.2/− 1.5	− 0.5/− 1.0

*NW* nanowire, *NS* nanosheet, *W*_*NS*_ width of nanosheets, *D*_*NW*_ diameter of nanowires; *All SS values were estimated with V_DS_ being at saturation regime.

**Figure 8:**
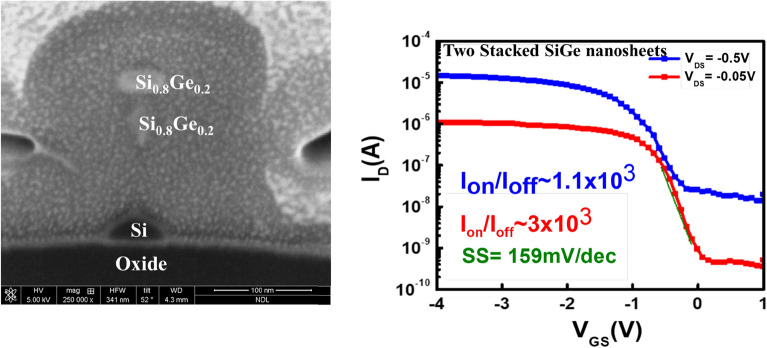
I_D_–V_GS_ for the Si_0.8_Ge_0.2_ nanosheets after selective wet etching of Si sacrificial layers (not optimized).

We found there would be a remaining Si parasitic channel underneath the Si_0.8_Ge_0.2_ NS and the shape of SiGe nanosheet would distort if the process of selective wet etching of Si sacrificial layers is not optimized (see Fig. 8), for instance, the temperature of TMAH solution is lower than 60 °C and concentration is less than 2.38%. The electrical measurement in Fig. 8b shows this parasitic Si channel influences the overall device performance by showing worse SS characteristic. Besides, the irregular shape of Si_0.8_Ge_0.2_ channel was observed to give relatively less overall perimeter compared to the rectangle nanosheet structure. Consequently, this will reduce the I_on_ current of the device.

## Conclusion

This study demonstrates the stacked Si_0.8_Ge_0.2_ NS p-FETs using Si/SiGe multilayers. Selective etching of Si over SiGe processes was successfully developed to obtain the Si_0.8_Ge_0.2_ nanosheets. The stacked SiGe NS GAAFETs have the potential to fulfill the requirement for the 3 nm technology node and beyond. Future studies will focus on further optimization of its performance terms of etch-selectivity and I_on_ current. The technique demonstrated in this study has a significant potential to boost the p-FET device performance for the next generation of CMOS logic in GAA NS technology.

## Methods

Si-on-insulator (SOI) wafers with a 40 nm thick Si top layer (p-type, 9–18 Ω cm) were employed as the substrates. The wafers were cleaned using the RCA standard cleaning methods for removing organic materials, certain metals, and particles from the Si substrates; the wafers were subsequently rinsed in deionized water and dried in N_2_ gas. Four alternative layer stacking of SiGe (60 nm) and Si (25 nm) were epitaxially grown on SOI (100) wafer with a 40 nm Si top seed layer. GeH_4_ and DCS gases were used to build the layer using an ASM Epsilon 2000 + low-pressure CVD machine. The growth temperature for the SiGe and Si layer was 700 °C. The growth pressure in chamber was kept at 20 torr. Electron-beam lithography and dry etching with Cl_2_/HBr were utilized to define and form the active device area, respectively. TMAH solution at 60 °C was used to remove Si interlayers selectively and form the stacked SiGe nanosheets. The gate dielectrics Y_2_O_3_ were formed by ALD^18^. The gate metal TiN was deposited by PVD sputtering.
